# Organic—Inorganic Hybrid Interfaces Enable the Preparation of Nitrogen-Doped Hollow Carbon Nanospheres as High-Performance Anodes for Lithium and Potassium-Ion Batteries

**DOI:** 10.3390/ma16144936

**Published:** 2023-07-11

**Authors:** Yao Dai, Dong-Chuan Mo, Zong-Tao Qu, Wen-Kang Wang, Shu-Shen Lyu

**Affiliations:** 1 School of Materials, Sun Yat-sen University, Shenzhen 51800, China; 2 Guangdong Engineering Technology Research Centre for Advanced Thermal Control Material and System Integration (ATCMSI), Sun Yat-sen University, Shenzhen 51800, China

**Keywords:** hybrid interface, rich nitrogen, hollow carbon nanosphere, lithium-ion batteries, potassium-ion batteries

## Abstract

An abundant hollow nanostructure is crucial for fast Li^+^ and K^+^ diffusion paths and sufficient electrolyte penetration, which creates a highly conductive network for ionic and electronic transport. In this study, we successfully developed a molecular-bridge-linked, organic–inorganic hybrid interface that enables the preparation of in situ nitrogen-doped hollow carbon nanospheres. Moreover, the prepared HCNSs, with high nitrogen content of up to 10.4%, feature homogeneous and regular morphologies. The resulting HCNSs exhibit excellent lithium and potassium storage properties when used as electrode materials. Specifically, the HCNS-800 electrode demonstrates a stable reversible discharge capacity of 642 mA h g^−1^ at 1000 mA g^−1^ after 500 cycles for LIBs. Similarly, the electrode maintains a discharge capacity of 205 mA h g^−1^ at 100 mA g^−1^ after 500 cycles for KIBs. Moreover, when coupled with a high-mass-loading LiFePO_4_ cathode to design full cells, the HCNS-800‖LiFePO_4_ cells provide a specific discharge capacity of 139 mA h g^−1^ at 0.1 C. These results indicate that the HCNS electrode has promising potential for use in high-energy and environmentally sustainable lithium-based and potassium-based batteries.

## 1. Introduction

Lithium-ion batteries (LIBs) have garnered extensive interest as a promising device for next-generation energy storage owing to their exceptional combination of high energy and power density [1,2,3]. Previous studies have shown that the electrochemical performance of LIBs with respect to energy and power density is majorly influenced by electrode materials comprising anode and cathode materials [4,5,6,7]. Potassium and lithium both belong to the first group of elements and possess comparable chemical and physical properties. An intriguing note is that the standard electrode potential of potassium is −2.93 V, which is close to the value of lithium. This indicates that potassium-ion batteries (KIBs) will have certain research value and application prospects [8,9]. Currently, carbonaceous materials are being investigated as suitable anode materials [10,11,12]. The development and application of carbon materials have greatly promoted the commercialization of LIBs, and graphite carbon materials still dominate in terms of the negative pole of commercial LIBs [13]. However, graphite carbon electrodes still exhibit certain drawbacks; for example, the diffusion path is too long for Li^+^ because of the anisotropy of graphite and smaller layer spacing, leading to a low specific capacity [14,15]. In contrast, amorphous carbon demonstrates superior performance for LIBs and KIBs with its larger average interlayer spacing that enables the accommodation of Li^+^ and K^+^ to adapt to the volume expansion [16,17,18,19].

Heteroatom doping is a successful strategy for improving the lithium and potassium storage capabilities of electrode materials by optimizing their electronic structure and strengthening their electrochemical performance [20]. Nitrogen, sulfur, boron, and phosphorus are common heteroatoms that have been extensively studied when doped into carbon materials [12,21,22,23,24]. Nitrogen doping, in particular, has been the most widely investigated due to the chemical similarity and close atomic radii between nitrogen and carbon atoms, leading to a facile doping process with high efficiency [25,26,27]. Among the various nitrogen-doped carbon materials, pyrrole-N and pyridine-N have been found to induce more defects and vacancies, thereby increasing the number of active sites [28]. Hollow carbon nanospheres (HCNSs) have been used as anode materials due to their unique properties such as high surface area, adjustable porosity, and superior mechanical strength [29]. The hollow structure of an HCNS provides a “buffer zone” that accommodates volume variations during charge and discharge, while also decreasing the ion diffusion distance [30]. Furthermore, carbon may properly improve a substance’s electrical conductivity, which is a crucial factor in electrode materials [31,32].

Herein, we synthesized in situ HCNSs with exceptional nitrogen content of up to 10.4%. The synthesis method involved the surface modification of silica nanospheres with silane coupling agents to facilitate the in situ surface-polymerization of pyrrole. Furthermore, the HCNS electrode showed remarkable lithium and potassium storage capabilities with superior cycling stability and high capacity. Our research provides a realistic route for tailoring Li^+^ and K^+^ storage performance by using heteroatom doping, and it opens the door for the use of disordered carbon materials in the fascinating field of chemistry and materials science.

## 2. Experimental Section

### 2.1. Synthesis of HCNS

The SiO_2_ nanospheres and SiO_2_-NH_2_@PPy were prepared through a facile method described earlier [33]. A solution of 70 mL of ethanol, 6 mL of ammonia solution (28 wt.%), and 4 mL of distilled water was typically put into a 250 mL flask with magnetic swirling, and a combination containing 6 mL of TEOS and 40 mL of ethanol was dropwise added to the as-prepared solution under fast stirring at 30 °C for 4 h. The resultant SiO_2_ nanospheres were centrifuged out of the suspension and cleaned ultrasonically with distilled water and ethanol.

A combined solution of 30 mL of distilled water and 30 mL of ethanol included 800 mg of SiO_2_ nanospheres that was evenly disseminated throughout the mixture. The aforesaid mixture was stirred for 15 min at room temperature before 0.4 mL of KH550 was added dropwise and maintained ultrasonically for 90 min, resulting in the creation of SiO_2_-NH_2_ nanospheres. Then, after 30 min of continuous stirring, 0.8 mL of pyrrole monomers was added to the combined solution in the ice-water bath. After that, 20 mL of 0.12 M K_2_S_2_O_8_ solution was dropwise added to the aforementioned solution at a rate of 0.5 mL per minute while being stirred magnetically for 12 h. Following the reaction, the SiO_2_-NH_2_@PPy sample was separated via centrifugation and repeatedly rinsed with diluted water.

The SiO_2_-NH_2_@PPy sample was subjected to heat treatment at 800 °C for 2 h in a nitrogen atmosphere. Black powder was obtained by etching the SiO_2_ template of SiO_2_-NH_2_@C in a 4 M NaOH solution, centrifugation, and washing with distilled water. The preparation processes for HCNS-600 and HCNS-1000 were identical to that of HCNS-800, except for the calcination temperatures, which were set to 600 °C and 1000 °C, respectively.

### 2.2. Characterization

The surface properties and crystal structures of HCNS samples were analyzed via X-ray photoelectron spectroscopy (XPS, ESCALAB 250, Thermo-VG Scientific, Waltham, MA, USA) and X-ray diffraction (XRD) with a Rigaku (D-max 2200VPC, Tokyo, Japan). The morphologies of the HCNS samples were examined using a scanning electron microscope (SEM, FEI-Q400F, Thermo-VG Scientific, USA) and a transmission electron microscope (TEM, JEOL-2010 HR, JEOL Ltd., Akishima, Japan). The BET specific surface areas of the HCNS samples were determined via nitrogen adsorption using a Micromeritics ASAP 2460, Micromeritics instrument Ltd., Norcross, GA, USA.

### 2.3. Electrochemical Measurements

Coin-type 2032 cells were utilized to conduct electrochemical measurements. The CR2032 coin-type cells were assembled in a glove box filled with Ar gas. The working electrode was prepared through the grinding of a mixture of active components, carbon black, and polyvinylidene fluoride (PVDF) in a weight ratio of HCNS/carbon black/PVDF = 8:1:1 to form a slurry. The slurry was then cast onto a copper foil. For LIBs, lithium foil served as the counter-electrode, and 1 M LiPF_6_ was utilized as the electrolyte in a mixed solution of dimethyl carbonate (DMC) and ethylene carbonate (EC). The counter-electrode for KIBs was potassium foil, and the electrolyte was a 0.8 M KPF_6_ solution in a mixture of DMC and EC. The Neware battery testing equipment was used to record the cycle performances with the potential range of 0.01–3.00 V for half cells and 2.2–3.6 V for full cells. The CV and EIS were measured using a Chenhua electrochemical workstation (CHI760E). The potential scan rate was set between 0.1 and 1.0 mVs^−1^, and the measurement was conducted from 0.01 to 3.00 V.

### 2.4. Molecular Dynamics Computational Methods

The total energy and energy change in the N doping system and N-P co-doping system were investigated using theoretical calculations. All structures were optimized with a convergence criterion of 1 × 10^−5^ eV for the energy and 5 × 10^−3^ eV/Å for the forces. Density functional theory (DFT) was used to carry out the theoretical computations. Geometric optimizations were performed using the Perdew–Becke–Ernzerhof (PBE) functional.

## 3. Results and Discussion

**Figure 1:**
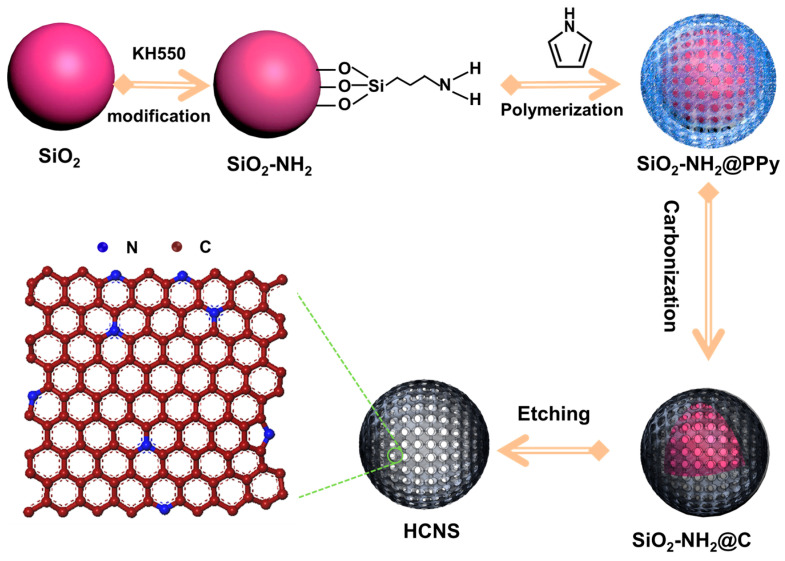
The preparation process of HCNSs.

**Figure 2:**
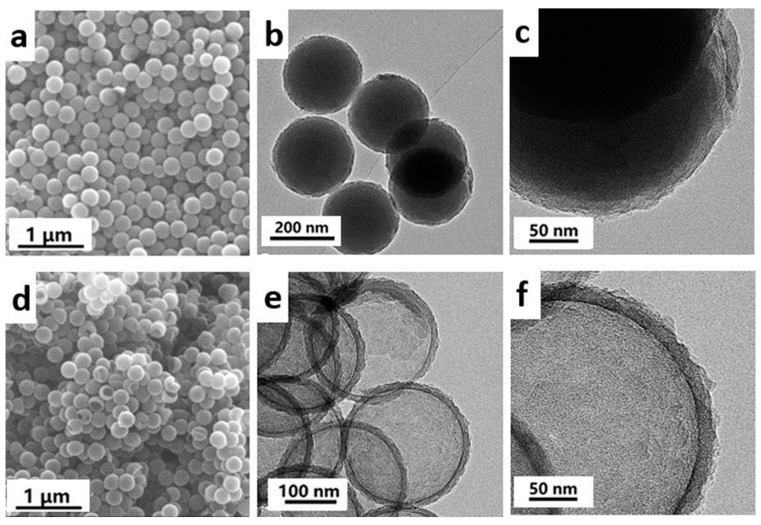
(**a**) SEM and (**b**,**c**) TEM images of SiO_2_-NH_2_@C; (**d**) SEM and (**e**,**f**) TEM images of HCNS-800.

Figure 1 shows the process for preparing HCNS. The SiO_2_ was synthesized using the Stöber method and modified with KH550 to enable the in situ surface-polymerization of pyrrole [33]. After calcination and hot alkali etching to remove the silica, HCNSs were obtained. Figure 2 shows the morphology and size of the resulting HCNS-800. The SiO_2_ template had an average diameter of 220 nm (Figure S1). The SiO_2_-NH_2_@C composite had a roughened core–shell morphology (Figure 2a–c and Figure S2) after polypyrrole coating and carbonization. Following the etching of the silica template, HCNS-800 was produced, displaying a flawless hollow nanosphere structure with dimensions of about 250 nm for the outer diameter, 220 nm for the hollow core, and 15 nm for the shell thickness (Figure 2d–f).

**Figure 3:**
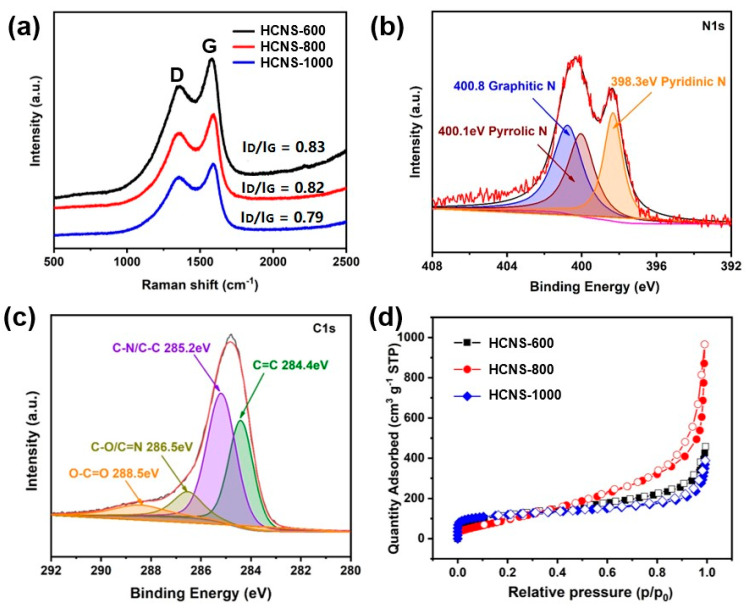
(**a**) Raman spectra of as-prepared samples of HCNS-600, HCNS-800, and HCNS-1000; (**b**) N1s and (**c**) C1s high-resolution XPS spectra of HCNS-800; (**d**) nitrogen adsorption–desorption isotherms of HCNS-600, HCNS-800, and HCNS-1000.

The XRD patterns of HCNS-600, HCNS-800, and HCNS-1000 are shown in Figure S3. It can be observed that all three samples exhibited two broad peaks at approximately 26° and 44°, nicely corresponding to the (002) and (101) planes of amorphous carbon [34]. All of the samples presented weaker peaks with a slight shift to the left compared with the graphite, possibly due to the increase in interlayer spacing with the introduction of nitrogen. The (002) plane interlayer spaces of the three HCNS samples, summarized in Table S1, were larger than the pristine graphite (0.336 nm), which was beneficial for enhancing the insertion process of K^+^ and Li^+^ [35]. The Raman spectra (Figure 3a) of the HCNS exhibited a significant difference with the change in calcination temperature. The ratio (I_D_/I_G_) of the D peak intensity at 1340 cm^−1^ to the G peak intensity at 1580 cm^−1^ was used to assess the degree of disorder in the carbon components [16,36]. The value for HCNS-600 (0.83) was close to HCNS-800 (0.82) but higher than that of HCNS-1000 (0.79). These results indicate that a lower calcination temperature can result in a more disorganized structure in the carbon material [37].

XPS analysis of the HCNS-800 sample indicated the presence of C, N, and O (Figure S4). The N content percentages in HCNS-600, HCNS-800, and HCNS-1000 were reported to be 10.4%, 5.6%, and 5.8%, respectively, and can be found in Table S1 [38]. The N1s high-resolution spectrum of the HCNS-800 displayed three peaks for pyridinic-N, pyrrolic-N, and graphite-N, respectively, at 398.3, 400.1, and 400.8 eV, as can be seen in Figure 3b [39]. Figure 3c shows four peaks that were assigned to C=C, C-N, C-O, and O-C=O, respectively, at 284.4, 285.2, 286.5, and 288.5 eV [40]. Figure 3d presents the adsorption–desorption isotherm curve of HCNS; micropores were present, since there was an adsorption uptake at low relative pressure. The specific surface area of HCNS-800 was 721 m^2^ g^−1^, which was higher than those of HCNS-600 (405 m^2^ g^−1^) and HCNS-1000 (414 m^2^ g^−1^), as summarized in Table S1. The abundant microporous structure of HCNS-800 facilitated quick ion diffusion routes and electrolyte penetration, which enhanced the electrode’s electrochemical performance [41,42,43].

**Figure 4:**
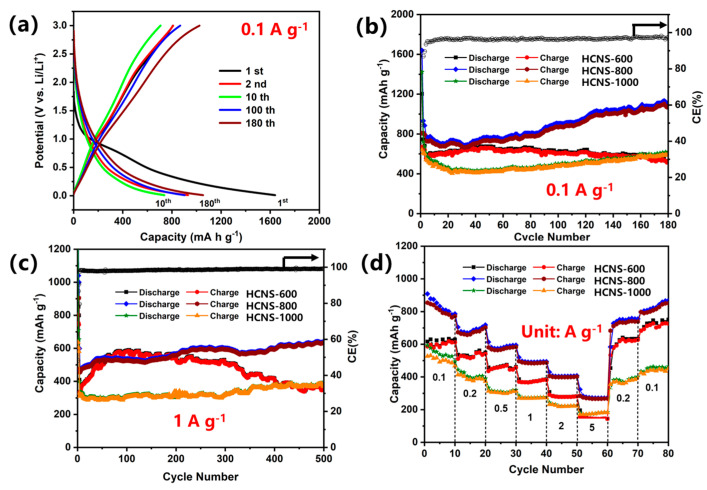
The HCNSs as anode materials for LIBs: (**a**) charge–discharge voltage profiles of HCNS-800 electrode; (**b**) and (**c**) cycling performances and coulombic efficiency (CE); (**d**) rate performance of HCNS-600, HCNS-800, and HCNS-1000 electrodes.

**Figure 5:**
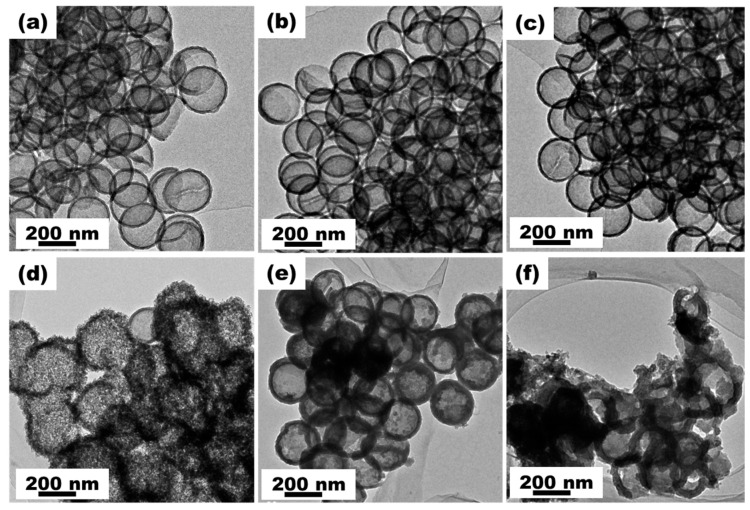
TEM images of (**a**) HCNS-600, (**b**) HCNS-800, and (**c**) HCNS-1000 electrodes before cycling; (**d**) HCNS-600, (**e**) HCNS-800, and (**f**) HCNS-1000 electrodes after cycling for LIBs.

The electrochemical properties of the HCNS electrodes were evaluated using a 2032 half-cell. The galvanostatic charge–discharge profiles (Figure 4a) showed that the first discharge–charge capacities of the HCNS-800 electrode were 1640.0 mA h g^−1^ and 807.6 mA h g^−1^, respectively. The consumption of SEI development between the electrode surfaces may have been the cause of the irreversible capacity [36]. The HCNS-800 electrode retained a discharge capacity of 1124.0 mA h g^−1^ after 180 cycles, higher than the HCNS-600 (528.6 mA h g^−1^) and HCNS-1000 (618.6 mA h g^−1^), as shown in Figure 4b. The HCNS-800 electrode was also superior to SiO_2_ and SiO_2_-NH_2_@C (Figure S5), as well as other carbon-based anode materials (Table S2). Furthermore, the HCNS-800 electrode delivered a reversible capacity of 642.4 mA h g^−1^ at 1 A g^−1^ after 500 cycles, higher than those of the SiO_2_ and SiO_2_-NH_2_@C samples (Figure 4c and Figure S6). The capacity was also 1.8 times that of the HCNS-600 electrode (359.1 mA h g^−1^) and 1.9 times that of the HCNS-1000 electrode (339.2 mA h g^−1^). The discharge capacities of the HCNS-800 electrode at increasing current rates of 0.1, 0.2, 0.5, 1, 2, and 5 A g^−1^ were found to be 832, 685, 575, 494, 404, and 270 mA h g^−1^, respectively; the reversible capacity of the electrode could be restored to 858 mA h g^−1^ when the current was reverted to 0.1 A g^−1^, as presented in Figure 4d. The rate performance was better than those of the HCNS-600, HCNS-1000, SiO_2_, and SiO_2_-NH_2_@C (Figure S7). Figure 5 presents the TEM images of HCNS electrodes before cycles and after cycles at a current density of 1 A g^−1^. All the HCNS electrodes demonstrated a perfect hollow nanosphere structure before cycles (Figure 5a–c). The HCNS-600 (Figure 5d) and HCNS-1000 (Figure 5f) electrodes showed a large amount of breakage due to the carbon sphere structure cracking during the repeated lithium insertion and desertion processes, whereas HCNS-800 could maintain its original morphology even after a long cycle process, as shown in Figure 5e. Therefore, the HCNS-800 sample exhibited superior cycling stability compared to the other two HCNSs.

**Figure 6:**
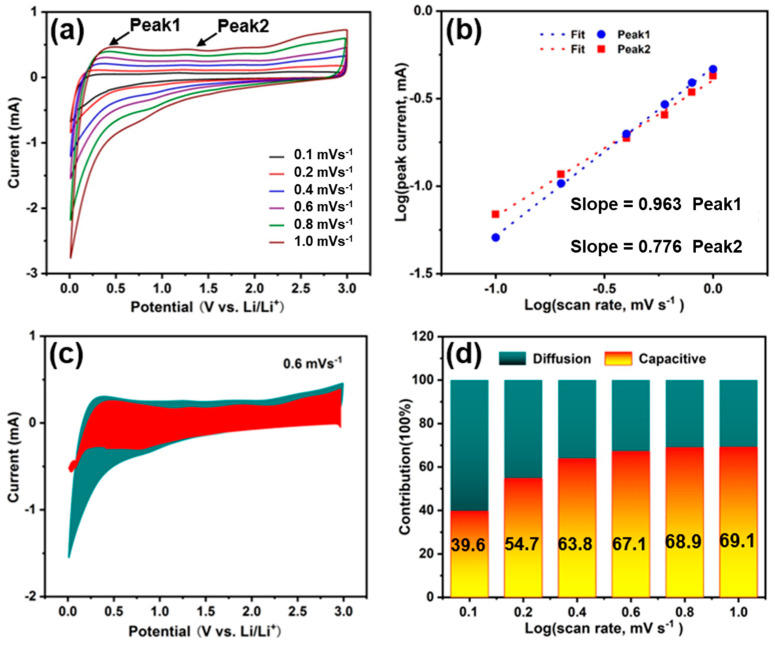
(**a**) The CV curves of the HCNS-800 electrode at various scan rates (0.1, 0.2, 0.4, 0.6, 0.8, and 1.0 mVs^−1^); (**b**) plots of log(i) versus log(v) at Peak1 and Peak2; (**c**) capacitive (red) and diffusion-controlled (cyan) contribution at 0.6 mVs^−1^; (**d**) the proportion contribution of capacitive and diffusion-controlled capacities at various scan rates for LIBs.

The diffusion contributions for the HCNS-600, HCNS-800, and HCNS-1000 electrodes were explored to explain the favorable Li^+^ storage performance. Figure 6a and Figure S8 depict the CV curves obtained at five different scan rates (0.1, 0.2, 0.4, 0.6, 0.8, and 1.0 mV s^−1^) and display a well-progressive shape. The following equations govern the current (i) and scan rate (v) of CV curves, as is well known [44]:

(1) $$i=av^{b}$$

(2) log(⁡i)=blog(⁡v)+log⁡a

A b-value of 0.5 indicates a diffusion-controlled process, whereas a *b*-value of 1 indicates a pseudocapacitive impact [45,46]. Figure 6b shows that the b values are 0.963 and 0.776, corresponding to peak 1 and peak 2, respectively, indicating the storage of potassium in HCNS-800 involves a diffusion and pseudocapacitive mechanism. This indicates that the Li-ion storage is mainly controlled by pseudocapacitive reactions, resulting in fast reaction kinetics [47]. Following that, capacitive contributions were evaluated [48].

(3) ⅈ=k1ν+k2v12

Figure 6c shows the capacitive capacity contribution ratio (red region) of the HCNS-800 electrode was approximately 67.1% of the total capacity at a scan rate of 0.6 mV s^−1^. Figure 6d indicates that the contribution percentages were 39.6%, 54.7%, 63.8%, 67.1%, 68.9%, and 69.1% at scan speeds of 0.1, 0.2, 0.4, 0.6, 0.8, and 1.0 mVs^−1^, respectively, almost all higher than the values of HCNS-600 and HCNS-1000 electrodes (Figure S8), suggesting that the pseudocapacitive capacity played a significant role in the total capacity [25]. Therefore, the high capacitive capacity contribution of the HCNS-800 electrode may be one of the reasons for its favorable lithium storage performance [49]. EIS analyses of the HCNS samples after 500 cycles at 1 A g^−1^ are presented in Figure S9. The highest conductivity and lowest charge-transfer resistance R_ct_ values (34.4 Ω) were found in HCNS-800, compared to HCNS-600 (70.0 Ω) and HCNS-1000 (191.6 Ω), as shown in Table S3. Furthermore, the lithium-ion diffusion coefficients (*D_Li_*, cm^2^ s^−1^) of the three electrodes were calculated using Equation (4) [50].

(4) $$D_{Li}=\frac{1}{2}{\left[(\frac{V_{m}}{FA\sigma_{w}})\frac{dE}{dx}\right]}^{2}$$

The accurate diffusion coefficients of Li^+^ of HCNS-600, HCNS-800, and HCNS-1000 were calculated as 1.43 × 10^−9^, 3.57 × 10^−9^, and 6.21 × 10^−10^ cm^−2^ s^−1^, respectively. The *D_Li_* value of the HCNS-800 electrode was found to be about 2.5 times greater than that of the HCNS-600 and about 5.7 times greater than that of the HCNS-1000, as shown in Table S3.

**Figure 7:**
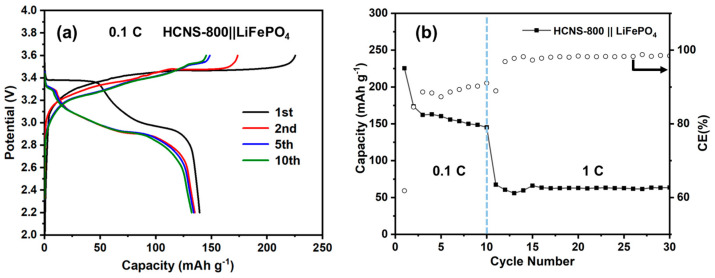
Electrochemical behavior of HCNS-800||LiFePO_4_ full cell in the voltage range of 2.2–3.6 V. (**a**) Galvanostatic charge–discharge profile of the cell at 0.1 C and (**b**) cyclic performance and CE at 1 C (1 C = 170 mA g^−1^).

In addition to half-cell analysis, the real use of HCNS-800 in LIBs was tested in the full-cell configuration with high-mass LFP loading (8.2 mg cm^−2^). Figure 7a discloses the cycling performance and charge–discharge behavior of the HCNS-800||LiFePO_4_ system. The full cell demonstrates the first charge–discharge capacities were 225.3 mA h g^−1^ and 139.3 mA h g^−1^, respectively. Moreover, the full cell showed an ideal discharge capacity of 139 mA h g^−1^ for the initial 10 cycles at 0.1 C and 61 mA h g^−1^ for the next 20 cycles at 1 C, as shown in Figure 7b. Theoretical calculations were performed to calculate the lithium-ion-binding energy for N-doped HCNS combined structural models to gain a deeper understanding of their electrochemical performance in LIBs. The results of the DFT calculations showed that the N-doped HCNS and non-N-doped HCNS had ΔE_a_ values of −3.91 eV and −3.16 eV, respectively, as shown in Figure S10. The N-doped site was found to have a more negative ΔE_a_ value, indicating that it contributes to a higher Li-ion storage ability. These calculated results are in agreement with the actual test results.

**Figure 8:**
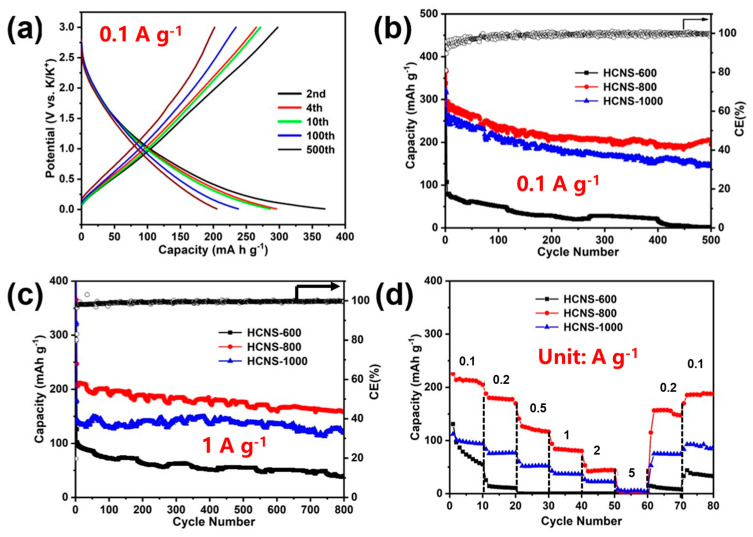
The HCNSs as anode materials for KIBs: (**a**) Charge–discharge voltage profiles of the HCNS-800 electrode at a current density of 0.1 A g^−1^. (**b**) Cycling performance at 0.1 A g^−1^ and (**c**) at 1 A g^−1^ and (**d**) rate capability of the HCNS-600, HCNS-800 and HCNS-1000 electrodes.

The electrochemical performance of HCNSs as anode materials for KIBs was investigated. The galvanostatic charge–discharge voltage profiles of the HCNS-800 electrode at 0.1 Ag^−1^ are presented in Figure 8a. The second discharge–charge capacities of the HCNS-800 electrode were 369.0 mA h g^−1^ and 297.7 mA h g^−1^, respectively. The reversible capacity remained stable over the charge–discharge cycles after 100 cycles, and the electrode’s discharge capacity could be maintained at 205.6 mA h g^−1^ after 500 cycles, outperforming the other electrodes, as shown in Figure 8b. As demonstrated in Figure 8c, the HCNS-800 electrode exhibited excellent reliability and stability with 143.5 mA h g^−1^ at 1 A g^−1^ after 1000 cycles. Additionally, the comparative rate performances of the three electrodes at different current densities from 0.1 to 5 Ag^−1^ are presented in Figure 8d. The electrode delivered average discharge capacities of 212, 177, 123, 82, and 43 mA h g^−1^ at current densities of 0.1, 0.2, 0.5, 1, and 2 Ag^−1^, respectively. Notably, when the current density returned to 0.1 A g^−1^ after high-rate testing, the HCNS-800 electrode recovered to 187 mA h g^−1^, indicating fast kinetics and good stability. In contrast, the other electrodes exhibited much lower capacities at each corresponding current density. The excellent K^+^ storage performance for the HCNS-800 electrode was investigated in terms of the diffusion contribution. The CV curves are shown in Figure S11 and have well-progressive shapes. At a scan rate of 0.6 mV s^−1^, the capacitive capacity contribution ratio of the HCNS-800 electrode was roughly 65.2% of the overall capacity. At scan speeds of 0.1, 0.2, 0.4, 0.6, 0.8, and 1.0 mVs^−1^, the contribution percentages were 47.4%, 51.7%, 57.6%, 65.2%, 69.2%, and 80.1%, respectively, indicating that the pseudocapacitive capacity contributed significantly to the total capacity.

## 4. Conclusions

In summary, we successfully propose a well-orchestrated, molecular-bridge-linked, organic–inorganic hybrid interface for the preparation of core–shell composites and in situ nitrogen-doped hollow carbon nanospheres. Meanwhile, the resulting HCNS-800 exhibited excellent lithium and potassium storage properties as an electrode material, which can be attributed to the following merits. Firstly, the abundant microporous and mesoporous structure enables sufficient electrolyte penetration and helps create fast ion diffusion paths, thereby building a highly conductive network for electronic and ionic transport; secondly, the amorphous carbon with expanded interlayer distance and substantial defects can serve as the reservoir for sufficient lithium and potassium storage. Finally, the pseudocapacitive capacity contribution may be partially responsible for the excellent lithium and potassium storage performance. The molecular-bridge-linked, organic–inorganic hybrid interface will contribute to the production of well-defined nano/micro-structured carbon-based materials, and it also holds great promise for advancing the field of energy storage and enabling the creation of innovative and efficient energy storage solutions.

## Data Availability

The data is unavailable due to privacy.

## Supplementary Materials

The following supporting information can be downloaded at: https://www.mdpi.com/article/10.3390/ma16144936/s1, References [51,52,53,54,55,56,57,58,59,60,61,62] are cited in the supplementary materials.
