# Supplementary material for: Organic—Inorganic Hybrid Interfaces Enable the Preparation of Nitrogen-Doped Hollow Carbon Nanospheres as High-Performance Anodes for Lithium and Potassium-Ion Batteries
Source: Materials (Basel). 2023 Jul 11;16(14):4936. doi: 10.3390/ma16144936 (PMC10381384; doi:10.3390/ma16144936)
Supplement: Supplementary file 1 [file materials-16-04936-s001.zip › materials-2449165-supplementary.pdf]

Supporting information

Organic-Inorganic Hybrid Interface Enables the Preparation of Nitrogen-Doped Hollow Carbon Nanospheres as High-Performance Anodes for Lithium and Potassium-Ion Batteries

Yao Dai [1,2], *Dong-Chuan Mo* [1,2], *Zong-Tao Qu* [1,2], *Wen-Kang Wang* [1,2], *Shu-Shen Lyu* [1,2*]

[1] School of materials, Sun Yat-sen University, Shenzhen 51800, China.

[2] Guangdong Engineering Technology Research Centre for Advanced Thermal Control Material and System Integration (ATCMSI), Sun Yat-sen University, Shenzhen 51800, China.

* Corresponding author: lvshsh@mail.sysu.edu.cn

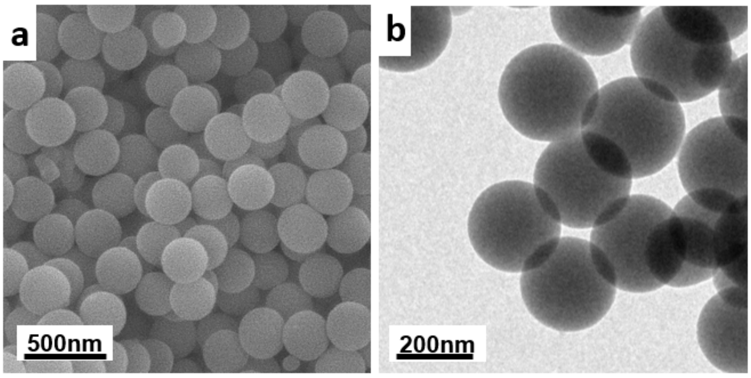

Figure S1 (a) SEM and (b) TEM image of SiO_2 nanospheres.

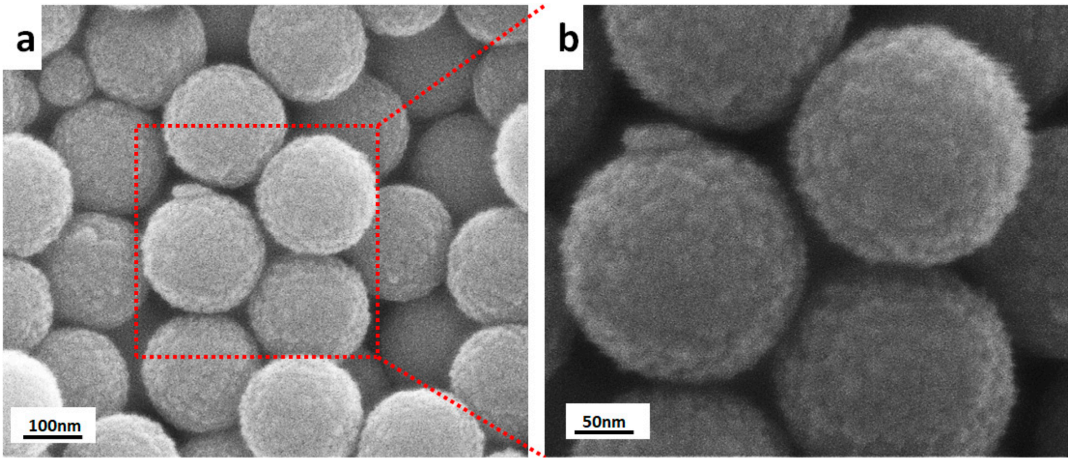

Figure S2 SEM image of SiO_2-NH_2@C.

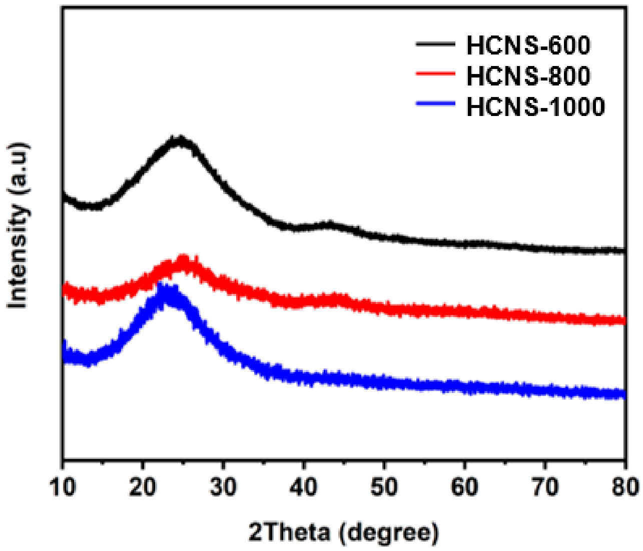

Figure S3 XRD patterns of HCNS with different calcination time.

Table S1 Physicochemical properties of HCNS samples.

Samples	S_{BET}[a]	I_D/I_G[b]	d_{002}[c]	C[d]	O[d]	N[d]
	m^2g^{-1}		nm	at%	at%	at%
HCNS-600	405	0.83	0.385	78.2	11.4	10.4
HCNS-800	712	0.82	0.378	85.4	9.0	5.6
HCNS-1000	414	0.79	0.372	89.1	5.1	5.8

[a] S_{BET} specific surface area

[b] the intense ratio of D band and G from Raman spectra

[c] interlayer distance of (002) plane calculated according to Bragg equation from XRD

[d] atomic percentage of the C, O and N elements of samples from XPS survey spectra

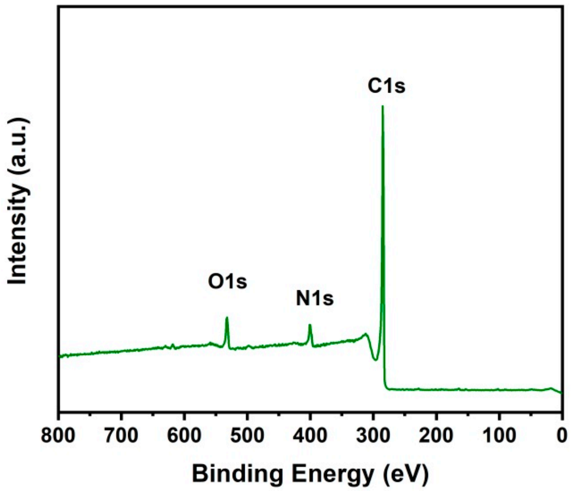

Figure S4 XPS spectra for the HCNS-800.

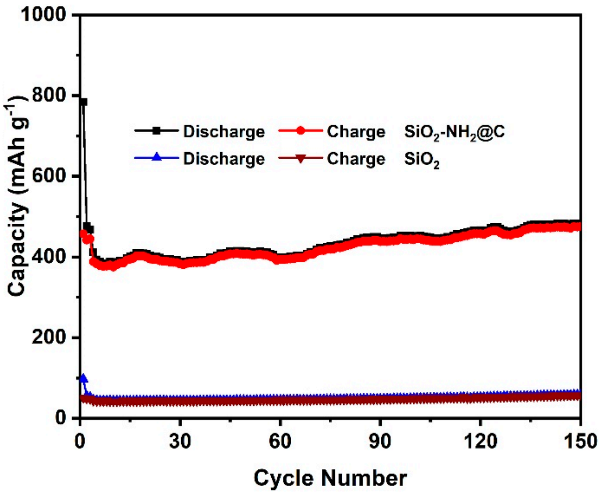

Figure S5 Cycling performances of SiO_2 and SiO_2-NH_2@C samples at 0.1Ag^{-1}.

Table S2 Comparison of LIBs performance for hollow structure and carbon-based anode materials

Samples	Current density (mA g^{-1})	Cycle number	Capacity (mAh g^{-1})	Reference
N-doped carbon nanotubes	1000	1800	343	[1]
N-doped porous carbon	200	500	450	[2]
N-doped porous carbon	100<br>500	300<br>500	597<br>513	[3]
Multi-shelled hollow Co_3O_4@C	1000	500	602	[4]
Porous graphene microsphere	2000	500	246	[5]
Hollow N-doped carbon nanobox	200	200	669	[6]
Hollow carbon nanoshperes	186	100	400	[7]
Hollow Carbon nanospheres	100<br>500	100<br>300	920<br>614	[8]
Hierarchical porous carbon microspheres	50	70	480	[9]
N-doped porous carbon microtubes	100	400	655	[10]
P-doped hollow porous carbon	1000	1000	359	[11]
Porous carbon nanofiber web	1000	100	549	[12]
N-doped hollow carbon spheres	100<br>1000	180<br>500	1124<br>642	This work

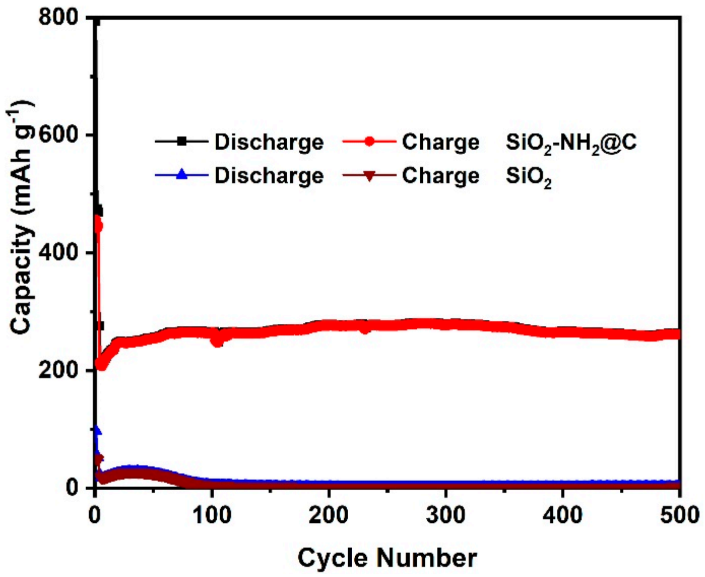

Figure S6 Cycling performances of SiO_2 and SiO_2-NH_2@C samples at $1Ag^{-1}$.

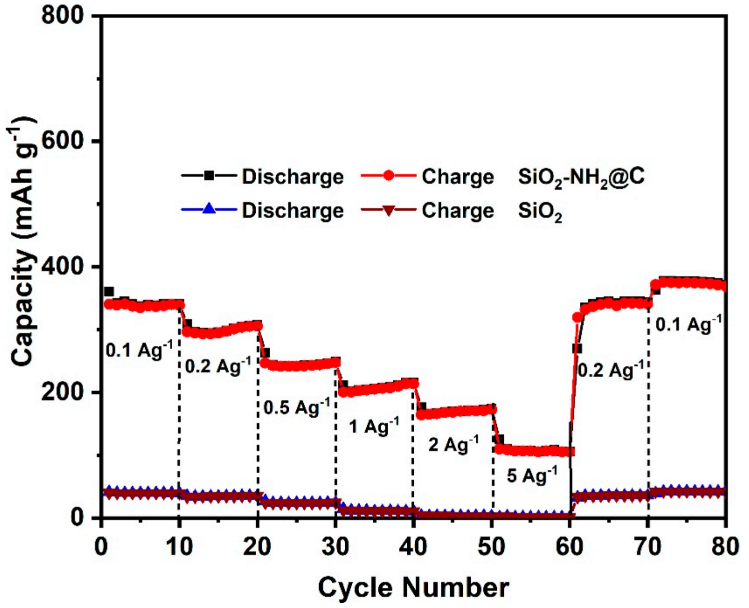

Figure S7 Rate performances of SiO_2 and SiO_2-NH_2@C samples.

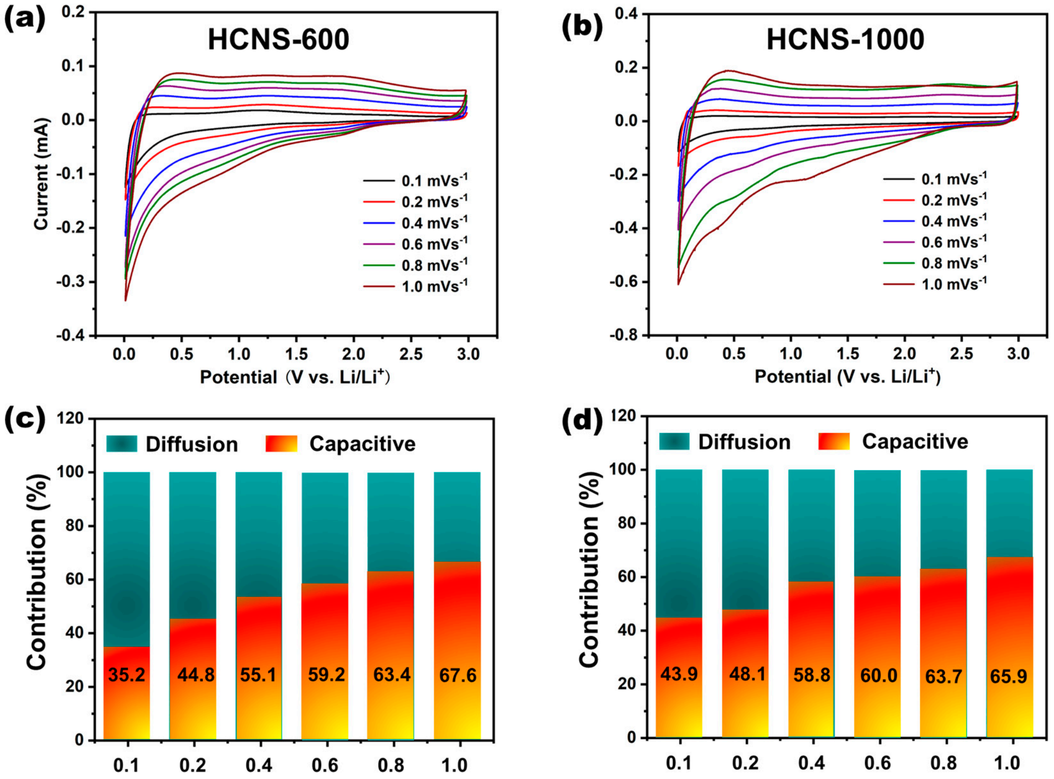

Figure S8 (a) CV curves of (a) HCNS-600 and (b) HCNS-1000 electrodes at various scan rates (0.1, 0.2, 0.4, 0.6, 0.8, and 1.0 mVs^{-1}); the proportion contribution of capacitive and diffusion-controlled capacities of (c) HCNS-600 and (d) HCNS-1000 electrodes at various scan rates.

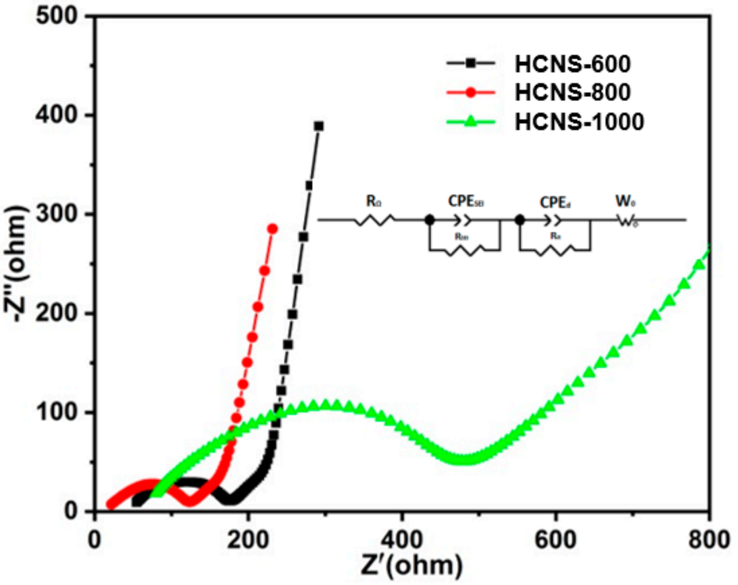

Figure S9 Electrochemical impedance spectroscopy (EIS) test of HCNS samples after

500 cycles at 1 Ag^{-1}.

Table S3 Physicochemical properties measured and calculated from the EIS spectra.

Samples	R_{SEI} (Ω)	R_{ct} (Ω)	D_{Li} ($cm^{-2}s^{-1}$)
HCNS-600	90.1	70.0	1.43×10^{-9}
HCNS-800	35.1	34.4	3.57×10^{-9}
HCNS-1000	193.4	191.6	6.21×10^{-10}

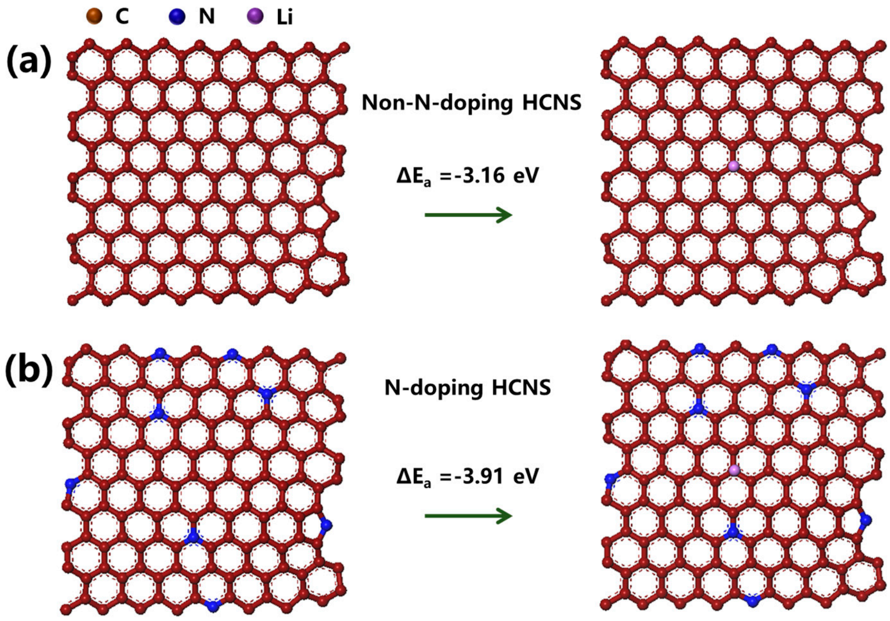

Figure S10 DFT calculations of the adsorption energies of lithium-ion on (a) non-N-doping HCNS and (b) N-doping HCNS.

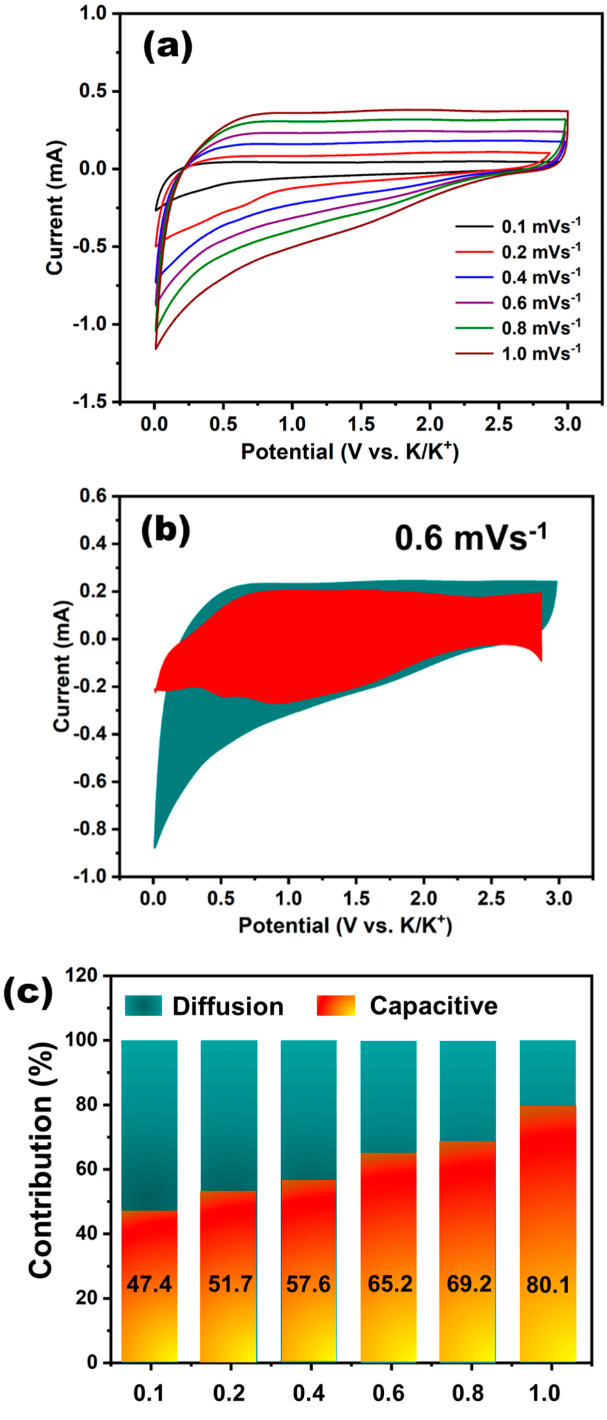

Figure S11 (a) The CV curves of HCNS-800 electrode at various scan rates (0.1, 0.2, 0.4, 0.6, 0.8, and 1.0 mVs^{-1}); (b) Capacitive (red) and diffusion-controlled (cyan) contribution at 0.6 mVs^{-1}; (c) the proportion contribution of capacitive and diffusion-controlled capacities at various scan rates for KIBs.

References

[1] S.Y. Zhong, H.Z. Liu, D.H. Wei, J. Hu, H. Zhang, H.S. Hou, M.X. Peng, G.H. Zhang, H.G. Duan, Long-aspect-ratio N-rich carbon nanotubes as anode material for sodium and lithium ion batteries, Chem Eng J 395 (2020) 125054.

[2] Z.H. Yan, Q.W. Yang, Q.H. Wang, J.M. Ma, Nitrogen doped porous carbon as excellent dual anodes for Li- and Na-ion batteries, Chinese Chem Lett 31 (2020) 583-588.

[3] J.G. Kim, H.C. Kim, N.D. Kim, M.S. Khil, N-doped hierarchical porous hollow carbon nanofibers based on PAN/PVP@SAN structure for high performance supercapacitor, Compos Part B-Eng 186 (2020) 107825.

[4] Y.C. Ding, L.H. Hu, D.C. He, Y.Q. Peng, Y.J. Niu, Z.Q. Li, X.X. Zhang, S.H. Chen, Design of multishell microsphere of transition metal oxides/carbon composites for lithium ion battery, Chem Eng J 380 (2020) 122489.

[5] B. Zhu, X.X. Liu, N. Li, C. Yang, T.Y. Ji, K. Yan, H.Y. Chi, X.L. Zhang, F. Sun, D.B. Sun, C.X. Chi, X. Wang, Y. Wang, L. Chen, L. Yao, Three-dimensional porous graphene microsphere for high-performance anode of lithium ion batteries, Surf Coat Tech 360 (2019) 232-237.

[6] T. Liang, H.W. Wang, R.X. Fei, R. Wang, B.B. He, Y.S. Gong, C.J. Yan, A high-power lithium-ion hybrid capacitor based on a hollow N-doped carbon nanobox anode and its porous analogue cathode, Nanoscale 11 (2019) 20715-20724.

[7] Q.L. Huang, S.L. Wang, Y. Zhang, B.W. Yu, L.Z. Hou, G. Su, S.S. Ma, J. Zou, H. Huang, Hollow Carbon Nanospheres with Extremely Small Size as Anode Material in Lithium-Ion Batteries with Outstanding Cycling Stability, J Phys Chem C 120 (2016) 3139-3144.

[8] C.P. Xu, D.C. Niu, N. Zheng, H.N. Yu, J.P. He, Y.S. Li, Facile Synthesis of Nitrogen-Doped Double-Shelled Hollow Mesoporous Carbon Nanospheres as High-Performance Anode Materials for Lithium Ion Batteries, Acs Sustain Chem Eng 6 (2018) 5999-6007.

[9] F.F. Wang, R.R. Song, H.H. Song, X.H. Chen, J.S. Zhou, Z.K. Ma, M.C. Li, Q. Lei, Simple synthesis of novel hierarchical porous carbon microspheres and their application to rechargeable lithium-ion batteries, Carbon 81 (2015) 314-321.

[10] H.G. Wang, C.P. Yuan, R. Zhou, Q. Duan, Y.H. Li, Self-sacrifice template formation of nitrogen-doped porous carbon microtubes towards high performance anode materials in lithium ion batteries, Chem Eng J 316 (2017) 1004-1010.

[11] S.J. Zhou, J. Li, L.C. Fu, J.J. Zhu, W.L. Yang, D.Y. Li, L.P. Zhou, Black Phosphorus/Hollow Porous Carbon for High Rate Performance Lithium-Ion Battery, Chemelectrochem 7 (2020) 2184-2189.

[12] W. Wang, Y. Sun, B. Liu, S.G. Wang, M.H. Cao, Porous carbon nanofiber webs derived from bacterial cellulose as an anode for high performance lithium ion batteries, Carbon 91 (2015) 56-65.
